# Role of complement factor B rs4151667 (L9H) polymorphisms and its interactional role with CFH Y402H and C3 rs2230199 (R102G) risk variants in age-related macular degeneration: a case control study

**DOI:** 10.1186/s12886-020-01552-4

**Published:** 2020-08-06

**Authors:** Nasrin Roshanipour, Maryam Ghaffari Laleh, Mortaza Bonyadi, Mohammad Hossein Jabbarpoor Bonyadi, Masoud Soheilian, Alireza Javadzadeh, Mehdi Yaseri

**Affiliations:** 1 Department of Biology, Tabriz Branch Islamic Azad University, Tabriz, Iran; 2 Center of Excellence for Biodiversity, Faculty of Natural Sciences, University of Tabriz, Tabriz, Iran; 3 Liver and Gastrointestinal Disease Research Center, Tabriz University of Medical Sciences, Tabriz, Iran; 4 Ocular Tissue Engineering Research Center, Ophthalmic Research Center, Shahid Beheshti University of Medical Sciences, Tehran, Iran; 5 Department of Ophthalmology, Tabriz University of Medical Sciences, Tabriz, Iran; 6 Department of Biostatistics and Epidemiology, Tehran University of Medical Sciences, Tehran, Iran

**Keywords:** Age-related macular degeneration (AMD), Complement factor B (CFB) gene, Complement factor H (CFH) gene, Complement factor 3 (C3) gene, L9H polymorphisms, Y402H polymorphism, R102G polymorphism, PCR-RFLP

## Abstract

**Background:**

Age-related Macular Degeneration (AMD) is a complex eye disease, which is genetically associated with different susceptibility loci. We planned to investigate the possible association of Complement Factor B (CFB) rs4151667 (L9H) variants and their possible interaction with Complement Factor H (CFH) Y402H and Complement factor 3 (C3) rs2230199 (R102G) in AMD.

**Methods:**

This case-control association study included 216 advanced type AMD patients and 191 healthy individuals for evaluation. Extracted-DNA samples were genotyped for the polymorphic regions of CFB rs4151667 (L9H), CFH Y402H and C3 rs2230199 (R102G).

**Results:**

The distribution of CFB rs4151667 (L9H) genotypes was not significantly different in the AMD patients compared to that of controls (*P* = 0.18). The AT genotype frequencies for CFB was non significantly lower in AMD group (6.5% vs. 13.1%, AOR = 0.49, CI = 0.23–1.04, *P* = 0.064(. The A allele of CFB rs4151667 (L9H) was found to be non-significantly lower in AMD patients. CFB rs4151667 (L9H) had no protective interactional effect against CFH (Y402H) and C3 (R102G) risk variants.

**Conclusions:**

This study showed that the protective role of CFB rs4151667 (L9H) in AMD is not significant and it has no significant protective interactional effect against CFH (Y402H) and C3 (R102G) risk variants.

## Background

Age-related Macular Degeneration (AMD) is a progressive neurodegenerative retinal disease and the leading cause of irreversible central vision in the elderly [1]. Aging is the main risk factor and recent evidence indicates the involvement of several environmental factors such as smoking in the pathogenesis of this disease [2]. Based on the pathological features of AMD, the role of dysregulation of inflammatory and immune responses in the etiology of the disease has been demonstrated [3–6]. As inflammation has the main role in the pathogenesis of AMD, dysfunction of the complement system is proposed to cause choroidal neovascularization [7, 8]. The role of the complement system was strengthened by evidence such as the expression of C3, C5, CFH, CFI and CFB genes in the retinal pigment epithelium (RPE) cells, and the presence of these proteins in drusen ​​structures [9–11]. Complement factor B (CFB) accelerates the initiation of the alternative complement cascade. Activation of this pathway is initiated by cleavage of C3b-bound factor B (BF), resulting in the formation of C3 convertase. Complement factor H (CFH), the major inhibitor of the alternative complement pathway imposes a regulatory role with C3 convertase dissociation [11]. A protective effect against the development of AMD has been reported for some polymorphisms of CFB in Caucasians [11–14]; however, this protective effect has not been seen in the Korean or Chinese population [15, 16].

In this case-control study, we aimed to investigate the possible role of CFB rs4151667 (L9H) polymorphism in Iranian patients with advanced AMD and to evaluate the interactional role of this locus polymorphism with CFH Y402H and C3 rs2230199 (R102G) risk variants.

## Methods

### Subjects

According to the inclusion and exclusion criteria described below, we evaluated 407 case-control samples consisting of 216 unrelated Iranian patients with AMD, and 191 genetically matched and unrelated Iranian controls without any signs of AMD. All AMD patients in at least one eye were referred from Labbafinejad Medical Center, Tehran; 22th Bahman Hospital, Gonabad; and Nikukari Eye Hospital, Tabriz.

The study was approved by the Ethics Committee of Ophthalmic Research Center, Shahid Beheshti University of Medical Sciences, Tehran, Ethical Board of Gonabad University of Medical Sciences, Gonabad, and the research ethics committee of Tabriz University of Medical Sciences, Tabriz. The present study was also performed in accordance with the Declaration of Helsinki and each participant in the study has filled out the written informed consent.

### Inclusion and exclusion criteria

Inclusion criteria included patients with the diagnosis of AMD, so that, presence of geographic atrophy or choroidal neovascularization with drusen more than five in at least one eye, and aged 50 years or older. Patients were excluded from study participation if they had retinal diseases other than AMD such as high myopia, retinal dystrophies, central serious retinopathy, vein occlusion, diabetic retinopathy, uveitis, and systemic inflammatory disease.

The control group in this study composed of individuals aged 50 years or older who were genetically matched and unrelated to the group of AMD patients with the absence of diagnostic criteria for AMD individuals with no drusen or retinal pigment epithelium changes- and absence of other retinal abnormality or systemic inflammatory disease.

### Diagnosis of AMD

A standard and comprehensive diagnosis of AMD is based on clinical examination or assessment of color fundus photographs. Measurement of vision acuity measurement, Slit-Lamp examination, and *dilated*-*pupil fundus* examination fundoscopy through a dilated pupil were done for all participants. Moreover, fluorescein angiography, Indocyanine Green angiography (ICG), and Optical Coherence Tomography (OCT) were performed, and diagnosis of AMD was confirmed.

AMD has several classification systems. AMD Usually classified into early and late stages. Early AMD stages according to traditional classifications include the presence of early or intermediate AMD according to the Beckman classification. Late AMD is defined by the presence of signs indicating either neovascular or atrophic AMD [4–6]. The cases were categorized based on the most severe optic disease. In the control subjects, there were no signs of macular pathology or early AMD such as drusen or irregular pigmentations of the RPE in the macular area.

### Genotyping

Genomic DNA was extracted from the controls and AMD patients’ blood using the YTA kit (No.YT9040-Favor Gen-Taiwan) as stated in the manufacturer’s instructions. For testing the rs4151667 (L9H) polymorphism, a pair of primers (Gen Fanavaran Co. Iran) were designed.

Primers used in this study were as follow: sense: 5′-AGTGATGTGGGTAGGACAGG-3′, antisense5’-TTGGAGAAGTCGGAAGGAGC-3″. To reach a volume of 25 ml of the reaction mixture, the reagents were added as follows: one microliter (100 ng) of genomic DNA, 5 μl Taq DNA Polymerase Master Mix RED (AMPLIQON- AMITIS GEN CO.) and 10 pmol of each primer. Setting up the conditions of thermal cycling were along these lines: a hot start denaturation step at 95 °C for 5 min, then 34 amplification cycles of denaturation at 94 °C for 30 s, annealing at 59 °C for 30 s, and elongation at 72 °C for 30 s, followed by a final extension at 72 °C for 5 min. The genotyping of CFB rs4151667 (L9H) was fulfilled by applying a suitable restriction enzyme, *BtsαI*. The *BtsαI* enzyme digests the amplicon, 456 bp, into two fragments of 222 bp and 234 bp for A allele, whereas the T allele remains uncut. All PCR products and restriction enzyme digested fragments were electrophoresis in a 2% agarose gel and visualized by gel red staining. C3 rs2230199 (R102G) and CFH Y402H genotyping method which have used for our patients and controls already has been published [4–6].

### Statistical analysis

Statistical tests were carried out using R software(R Core Team, 2015, R: A language and environment for statistical computing. R Foundation for Statistical Computing, Vienna, Austria. URL: http://www.R-project.org). The Categorical data between two groups were compared using chi-square test. To evaluate the Odds Ratio (OR) and Adjusted Odds Ratio (AOR) -considering the effect of age and sex- we used logistic regression. The *P*-value < 0.05 was considered statistically significant.

## Results

This case-control association study comprised 216 AMD patients (63.4% male) including 177 wet AMD and 39 advanced dry AMD, and 191 healthy individuals (53.9% male) (*P* = 0.052). The mean ± standard deviation (SD) age was 76 ± 8 years for AMD group and 73 ± 7 years in control group (*P* < 0.001). The baseline *characteristics* of AMD patients, its subgroups and controls are shown in Table 1. The frequencies of genotype distributions of all studied samples were in Hardy-Weinberg equilibrium (data not shown). The genotype and allele frequencies of the polymorphisms of the CFB rs4151667 (L9H) has been shown in Table 2. The comparison of frequency distribution in the genotypes was not significantly different between the AMD patients and that of controls (*P* = 0.18). The AT genotype frequencies for CFB was non-significantly lower in AMD group (6.5% vs. 13.1%, AOR = 0.49, CI = 0.23–1.04, *P* = 0.064(. Although the non-significant protective effect was maintained for wet type of AMD (AOR = 0.55; CI = 0.26–1.19; *P* = 0.13), this effect changes to a statistically significant risk effect in dry type of AMD (AOR = 1.20; CI = 1.12–1.28; *P* < 0.001) (Table 2). The A allele of CFB rs4151667 (L9H) was found to be non-significantly protective against AMD and its types (Table 2). The protective genotypes of CFB rs4151667 (non-TT) have protective interactional effect with CFH Y402H genotypes which is statistically significant only against non-risk CFH Y402H genotype, TT (OR = 0, *P* = 0.019, Table 3). The protective genotypes of CFB rs4151667 (non-TT) have protective interactional effect with C3 rs2230199 (R102G) genotypes which is statistically significant only against non-risk C3 rs2230199 (R102G) genotype, CC (OR = 0.44, *P* = 0.049, Table 3).

**Table 1:** Baseline features of study groups

		Total	Control	AMD			P1	P2	P3	P4
				Total	Wet	Dry				
**Age**	**Mean ± SD**	74 ± 7	73 ± 7	76 ± 8	75 ± 8	81 ± 7	< 0.001^a^	0.006^a^	< 0.001^a^	< 0.001^a^
**sex**	**Male**	240 (59.0%)	103 (53.9%)	137 (63.4%)	118 (66.7%)	19 (48.7%)	0.052^b^	0.013^b^	0.553^b^	0.035^b^
	**Female**	167 (41.0%)	88 (46.1%)	79 (36.6%)	59 (33.3%)	20 (51.3%)				

P1 comparison of AMD versus control, P2 comparison of wet AMD versus control, P3 comparison of dry AMD versus Control, P4 comparison of wet versus dry AMD

^a^ Based on t-test

^b^ Based on Chi-Square test

**Table 2:** Genotype and allele distribution of CFB (rs4151667) polymorphism among AMD patients and control group

		**Control**	**AMD**	**OR**	**95% CI**		**P**	**AOR**	**95% CI**		**P**
					Lower	Upper			Lower	Upper	
**Genotype**^a^							0.088				0.181
	TT	166 (86.9%)	201 (93.1%)	Ref				Ref			
	AT	25 (13.1%)	14 (6.5%)	0.46	0.23	0.92	0.028	0.49	0.23	1.04	0.064
	AA	0 (0.0%)	1 (0.5%)	–	–	–	–	–	–	–	–
**Allele**^b^	T	357 (93.5%)	416 (96.3%)	Ref				Ref			
	A	25 (6.5%)	16 (3.7%)	0.55	0.29	1.04	0.068	0.63	0.31	1.26	0.191
		**Control**	**Wet**	**OR**	**95% CI**		**P**	**AOR**	**95% CI**		**P**
					Lower	Upper			Lower	Upper	
**Genotype**^a^							0.146				0.320
	TT	166 (86.9%)	164 (92.7%)	Ref				Ref			
	AT	25 (13.1%)	12 (6.8%)	0.49	0.24	1.00	0.050	0.55	0.26	1.19	0.131
	AA	0 (0.0%)	1 (0.6%)	–	–	–	–	–	–	–	–
**Allele**^b^	T	357 (93.5%)	340 (96.0%)	Ref				Ref			
	A	25 (6.5%)	14 (4.0%)	0.59	0.30	1.15	0.121	0.70	0.35	1.42	0.324
		**Control**	**Dry**	**OR**	**95% CI**		**P**	**AOR**	**95% CI**		**P**
					Lower	Upper			Lower	Upper	
**Genotype**^a^							0.176				0.149
	TT	166 (86.9%)	37 (94.9%)	Ref				Ref			
	AT	25 (13.1%)	2 (5.1%)	0.36	0.08	1.58	0.176	1.20	1.12	1.28	**0.000**
	AA	0 (0.0%)	0 (0.0%)	–	–	–	–	–	–	–	–
**Allele**^b^	T	357 (93.5%)	76 (97.4%)	Ref				Ref			
	A	25 (6.5%)	2 (2.6%)	0.38	0.09	1.62	0.189	0.22	0.03	1.81	0.160
		**Dry**	**Wet**	**OR**	**95% CI**		**P**	**AOR**	**95% CI**		**P**
					Lower	Upper			Lower	Upper	
**Genotype**^a^							0.928				0.552
	TT	37 (94.9%)	164 (92.7%)	Ref				Ref			
	AT	2 (5.1%)	12 (6.8%)	1.35	0.29	6.31	0.700	3.28	0.39	27.66	0.275
	AA	0 (0.0%)	1 (0.6%)	–	–	–	–	–	–	–	–
**Allele**^b^	T	76 (97.4%)	340 (96.0%)	Ref				Ref			
	A	2 (2.6%)	14 (4.0%)	1.56	0.35	7.03	0.559	3.21	0.39	26.39	0.278

*OR* Odds ratio, *AOR* Adjusted Odds Ratio, consider the effect of age and sex

^a^ Based on logistic regression

^b^ Based on GLMM analysis

**Table 3:** Interaction of CFB (rs4151667) polymorphism genotypes with C3 (rs2230199)/CFH (Y402H) polymorphisms genotypes in AMD and control groups

	CFB	Case	Control	OR	95% CI		P
					Lower	Upper	
**C3** **Non-CC**	**Non-TT**	4	7	0.45	0.13	1.54	0.202
	**TT**	95	52	1.76	1.17	2.66	**0.007**
**CC**	**Non-TT**	10	17	0.44	0.20	1.00	**0.049**
	**TT**	126	111	0.79	0.54	1.17	0.238
**CFH** **Non-TT**	**Non-TT**	15	12	0.73	0.33	1.62	0.438
	**TT**	167	64	3.76	2.32	6.11	**0.000**
**TT**	**Non-TT**^a^	0	4	0.00	0.00	0.897	**0.019**
	**TT**	28	46	0.27	0.16	0.46	**0.000**

^a^ CI and P-value is calculated based on exact method and OR based on logistic regression. Discrepancy regarding the sample size in each combination is caused by missing data. C3 non-CC: risk genotypes of C3 rs2230199 (R102G), CFH Non-TT: risk genotypes of CFH Y402H

## Discussion

In this case-control study, which is the first report of the role of CFB rs4151667 (L9H) polymorphism in advanced AMD from this region, the possible interactional role of this locus with CFH Y402H and C3 rs2230199 (R102G) risk variants were studied. Our results showed neither the significant protective role of CFB rs4151667 (L9H) nor its interaction with other risk variants (Y402H and C3 rs2230199) in the development of AMD.

It should be bear in mind that the other genes of the complement system including C3 (rs2230199), CFI (rs141853578), and CFH (rs2274700, rs3753395, rs800292, and rs1061170) had previously studied in this population have shown their association with the disease [17–23].

In a meta-analysis, it has been shown that CFB rs4151667 (L9H) genotypes have protective effects against AMD [24] (pooled data: AA/TT OR = 0.99, AT/TT OR = 0.5 in Caucasians and AA/TT OR = 0.96, AT/TT OR = 0.68 in Asians). Bergeron-Sawitzke et al. [25] have reported a significant protective effect of this gene locus against AMD (AA+AT/TT OR = 0.39, *P* = 0.005, AA/TT OR = 0.48, AT/TT OR = 0.37). A protective effect for these polymorphisms of CFB against AMD has not been shown in Asians but has been found in some Caucasians [11–16, 24–28].

Inflammation plays a significant role in the pathogenesis of AMD, with CFB encoding protein that activates the complement pathway. Although the functional pathways of the genes involved in the complement pathway are related to AMD pathogenesis, we found no significant protective role of CFB rs4151667 (L9H) nor its interactional effect against Y402H and C3 rs2230199 (R102G) risk variants in AMD. In order to evaluate the result from a genetic association study in a population requires replication of that result in different populations. However, non-replication of this association in a population could be due to the presence of genetic diversities and/or the effect of other modifier genes in the studied phenotype(s) [29]. Precise elucidation of predisposing genetic factors to AMD in different populations could introduce future personalized therapeutic protocols.

## Conclusions

Our data could not demonstrate any significant association of the CFB rs4151667 (L9H) polymorphism with Age-related Macular Degeneration in Iranian patients. Also, there was not a significant protective interactional effect of this polymorphism against CFH (Y402H) and C3 (R102G) risk variants.

## Data Availability

The datasets used and/or analysed during the current study are available from the corresponding author on reasonable request.

## Abbreviations

AMD: Age-related Macular Degeneration
CFB: Complement Factor B
CFH: Complement Factor H
C3: Complement factor 3
RPE: Retinal Pigment Epithelium
ICG: IndoCyanine Green angiography
OCT: Optical Coherence Tomography
PCR: Polymerase Chain Reaction
OR: Odds Ratio
AOR: Adjusted Odds Ratio
CI: Confidence Interval
SD: Standard Deviation
